# The Therapeutic Potential of Different Surgical Approaches in the Management of Cardiac Myxoma: A Systematic Review

**DOI:** 10.3390/jcm14010121

**Published:** 2024-12-28

**Authors:** Dominik Mendyka, Tomasz Płonek, Tomasz Jędrasek, Adrian Korman, Aleksandra Złotowska, Aleksandra Jędrasek, Robert Skalik, Wojciech Kustrzycki

**Affiliations:** 1 Department of Cardiac Surgery, MEDINET Heart Center Ltd., 51-124 Wrocław, Poland; 2 Faculty of Medicine, Wroclaw Medical University, 50-367 Wrocław, Poland; 3 Department of Cardio-Thoracic Surgery, Thorax Centrum Twente Medisch Spectrum Twente, 7512 KZ Enschede, The Netherlands; 4 Faculty of Medicine, Wroclaw University of Science and Technology, 51-377 Wroclaw, Poland

**Keywords:** cardiac myxoma, management of cardiac myxoma, surgical treatment, minimally invasive approach, robotic surgery

## Abstract

**Background:** Cardiac myxomas are benign tumors of the heart. They occur mostly in the left atrium. The preferred treatment is surgical resection, which can be performed via conventional median sternotomy, minimally invasive, or robotic-assisted approaches. This study aimed to evaluate the outcomes, advantages, and limitations of these surgical methods, with a focus on their safety, efficacy, and impact on patient recovery. **Methods:** This systemic review was conducted according to PRISMA guidelines. The chosen databases were systematically searched using the keywords “cardiac myxoma”, “resection”, “approach”, “minimally invasive”, and “robotic surgery”. The comparison between the surgical strategies was based on thirteen articles, which met the inclusion criteria. **Results:** Median sternotomy remains the standard technique, providing excellent surgical access but is associated with longer hospital stays, higher blood loss, and increased risk of complications such as stroke. Minimally invasive approaches demonstrated comparable safety with shorter ICU stays, reduced blood loss, and lower postoperative pain but presented challenges, including limited exposure and longer procedure times. Robotic-assisted surgery showed potential as a safe alternative but was limited by high costs and technical demands. However, the data on minimally invasive and robotic methods are limited due to the rarity of cardiac myxomas. **Conclusions:** The choice of surgical technique should be individualised by considering the tumour size, location, patient condition, and surgeon expertise. Routine postoperative echocardiographic monitoring is essential to detect residual or recurrent tumours. Further studies are needed to validate the long-term efficacy of minimally invasive and robotic approaches.

## 1. Introduction

The heart is an organ in which tumours occur sporadically. Tumours arising in the heart can be primary or secondary [1,2]. Primary cardiac tumours occur in 0.3% of the population, with benign tumours making up 75% of these cases [2,3,4]. The most common primary cardiac tumours are myxomas, which account for over 50% of diagnoses [5]. In women, this figure rises to 75%, with the most common occurrence in women between the ages of 56 and 60 years [2,4,5,6,7]. Secondary cardiac tumours are metastases from extracardiac foci, occurring in 1.2% of the population [1,2].

The first description of a heart tumour was published by Realdo Colombo in 1559 [8]. In 1952, myxoma was first diagnosed by an angiography, performed by Henry Goldberg, though removal of the tumour was unsuccessful [9]. The introduction of cardiopulmonary bypass (CPB) by John Gibbon in 1953 allowed for safe access to the heart cavities [10]. As a result, Clarence Crafoord performed the first successful removal of a left atrial myxoma in 1954 [11]. In 1959, Kay successfully removed a left ventricular myxoma for the first time [12].

Cardiac myxomas are benign tumours of the heart, most commonly occurring in the left atrium (LA) [2]. These tumours typically have regular structures and smooth surfaces [13]. Their course is usually initially asymptomatic, making them primarily detected incidentally on cardiac imaging [14,15]. The presence of a myxoma may lead to serious systemic complications, such as heart failure or stroke, which is why prompt surgical intervention is crucial [16]. The method of choice for treating myxomas is complete resection of the tumour. The excision of myxomas can be performed using various methods, such as classical surgery (median sternotomy, MS), minimally invasive (MI) approaches (right anterolateral minithoracotomy, RAMT), or robotic-assisted intervention. Surgical advancements have significantly minimis ed the invasiveness of operations. MI surgery is mainly associated with fewer postoperative complications and shorter hospitalis ation times, but it also has certain limitations.

This systematic review summarises the current results of treating patients using different surgical methods, highlighting their advantages and limitations.

How do different surgical methods compare in terms of their advantages and limitations when treating patients, and what novel insights does this systematic review provide that address gaps in the existing literature?

## 2. Methodology and Search Strategy

**Figure 1:**
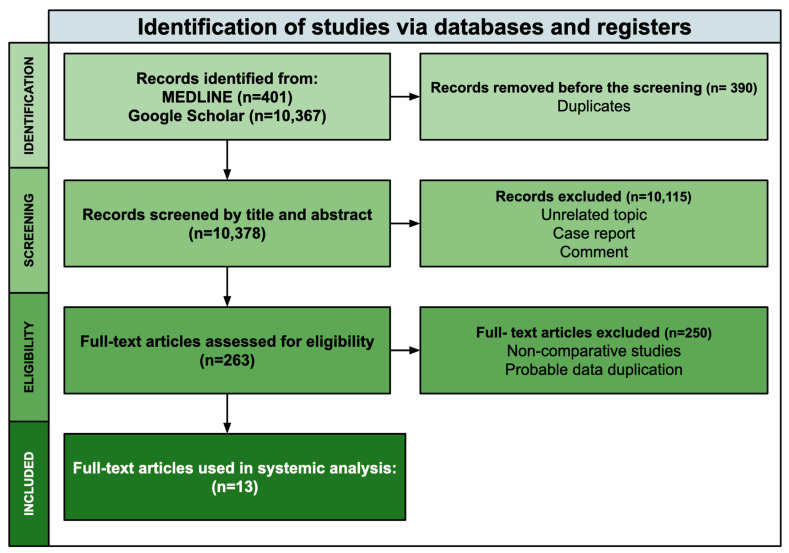
Literature search carried out according to the PRISMA guidelines.

This systematic review was performed following the PRISMA guidelines [17]. The MEDLINE and Google Scholar databases were systematically searched in November 2024 without any additional settings. The keywords used in the search included “cardiac myxoma,” “resection,” “approach,” “minimally invasive,” and “robotic surgery”. Only comparative studies were included. Case reports, comments and studies presenting the results of one technique were excluded. This systematic review acknowledges the potential for bias in the selection and interpretation of studies. Efforts were made to minimise it through a comprehensive search strategy, predefined inclusion criteria, and critical appraisal of the included studies. Three independent authors conducted the search (DM, TJ, and AK). Thirteen studies were included in the review. The search strategy is presented in Figure 1. Ethics approval was not required for this study.

## 3. Cardiac Myxoma

Cardiac myxomas are white, yellow, or brown tumours that range in size, reaching up to 15 cm and weighing up to 180 g [2,18]. The presence of vimentin in histochemical studies confirms their mesenchymal origin [4]. They are mostly polypoid, compact structures, often covered by a thrombus. In two-thirds of cases, they are oval in shape, with a smooth lobular surface [18]. Most are characterised by the presence of a pedicle, with a broad base in most cases. Myxomas rarely fragment spontaneously [5]. Echocardiography reveals a heterogeneous structure with areas of reduced echogenicity [2].

Cardiac myxomas most commonly appear in the second or third decade of life and are typically detected in the fourth or sixth decade [19]. Earlier diagnosis in younger individuals is often associated with differentiation from Carney syndrome, a genetic disorder inherited in an autosomal dominant manner, occurring in 10% of cases. In this syndrome, myxoid lesions occur in 20% of patients and are often multifocal, affecting both the atria and ventricles simultaneously [2,4,20]. Ten percent of patients with cardiac myxomas have a positive family history [18,19]. Myxomas have a 5% recurrence rate after resection [16,21].

Myxomas are 94% solitary, with some cases showing multifocal localisation. In 74% of cases, the tumour appears in the LA. The right atrium (RA) is the location in 10–20% (18%) of cases, with the remaining cases distributed evenly between the other heart chambers. In the LA, the tumour can arise anywhere, but 90% of cases occur at the interatrial septum, near the edge of the fossa ovalis, where it is connected by a pedicle. Studies also show other locations for myxomas, such as the free wall of the atrium, the appendage, the heart valves, the pulmonary artery, the pulmonary vein, and the vena cava [2,3,4,13,15].

### 3.1. Symptoms of Cardiac Myxoma

Initially, the presence of a tumour lesion does not cause any symptoms [22]. Subsequently, emerging symptoms can be considered uncharacteristic and can be divided into systemic and cardiovascular categories, depending on the location of the tumour.

Patients may present with febrile episodes, malaise combined with cachexia, joint pain, skin rash, and occasionally Raynaud’s sign [23,24]. One of the most common manifestations of myxoma is systemic embolism, which occurs in 30–40% of patients. It can be caused by a tumour fragment or thrombus from the tumour surface. The location of the embolism depends on the location of the tumour and intracardiac leaks [22,25,26]. The left-sided nature of most myxomas means that approximately 50% of embolic episodes affect the central nervous system, causing intracranial and extracranial emboli. Right-sided myxomas are responsible for pulmonary emboli [27,28].

Auscultation reveals a widely bifurcated first heart tone, the presence of a fourth tone, a holosystolic murmur, or a diastolic murmur due to blockage of the left atrioventricular outlet (which may vary with body position) [29]. Left atrial myxoma causes adventitious sounds on auscultation, including a diastolic mitral murmur (75% of patients), mitral regurgitation murmur (50% of patients), and third tone (33% of patients) [29]. When the tumour falls through the left atrioventricular orifice, a loud first heart tone can be heard due to a delayed mitral valve closure [29].

Right atrial myxoma most often manifests clinically with a picture of right ventricular (RV) failure, including hepatomegaly, ascites, and dependent oedema. In left ventricular (LV) myxoma, the condition can mimic subaortic or aortic valve stenosis, and in the RV, it may mimic pulmonary valve stenosis [18,22,23].

A summary of the symptoms and their frequency is presented in Table 1.

### 3.2. Diagnostic Imaging of Cardiac Myxoma

Echocardiography is the most commonly used diagnostic modality to diagnose cardiac masses, with a sensitivity of up to 100%. Its use enables the precise determination of the location, size, shape, and mobility of the tumour. It also allows for the assessment of the effect of the tumour on the cardiac flow and systolic parameters [2,4,14,30,31,32]. Transthoracic echocardiography is primarily used for evaluating larger tumours, mainly located in the LA. Transesophageal echocardiography (TEE) is preferred for smaller lesions and those located in unusual positions, such as the RA or the pulmonary trunk valve. For better visualisation of tumours on echocardiography, contrast agents can be used [32,33,34,35].

Echocardiography is an examination with high specificity and sensitivity for differentiating myxoma from thrombus. Thrombus typically shows homogeneous echogenicity or more intense reflections in the central zone [15,32,34,36]. Thrombus provides mobility, which depends on the length of the cap, the extent of attachment, and the number of collagen fibres in its structure. Within the tumour, focal areas of haemorrhage, necrosis, or cyst formation may also be revealed [15,18].

Additional examinations may include computed tomography (CT) or magnetic resonance imaging (MRI) scans. These modalities offer a larger imaging field and are primarily used when echocardiographic imaging provides insufficient data [20,32,34,35].

## 4. Surgical Techniques of Cardiac Myxoma Resection

### 4.1. Standard Approach (Median Sternotomy)

Surgery is the method of choice for treating myxomas, with median sternotomy being the most commonly used approach. It is especially preferred for large myxomas located in the atria or for tumours found in the ventricles [37,38]. Due to the significant fragility and tendency of myxomas to form blockages, manipulation of the heart should be minimis ed before starting extracorporeal circulation [39,40,41].

For myxomas located in the LA, both vena cavae are cannulated [42]. If extensive exposure of the LA is necessary, the superior vena cava is mobilised and directly cannulated, allowing it to be cut if additional exposure is needed [39,41,43]. The body temperature is allowed to fall to 32 °C [39,41,44]. Extracorporeal circulation is established, and a cardioplegic solution is administered according to local protocols [39,41]. Left atrial myxomas can be approached through an incision in the Waterston’s groove. This incision can be extended behind both veins for greater exposure [39,42,45,46]. By incising both atria, tumours attached to the fossa ovalis can be easily removed with full-thickness excision at the site of attachment, and the interatrial septum can be closed [38,39,41,45].

Right atrial (RA) myxomas cause special problems with venous cannulation. If the tumour is large or near both venous orifices, and classical cannulation is not possible, peripheral cannulation of the jugular and femoral veins can be used to initiate extracorporeal circulation [43]. After the aorta is cross-clamped and cardioplegia is administered, the RA is opened wide [42,46]. Resection of large or critically located RA myxomas often requires careful preoperative planning, intraoperative TEE, and special extracorporeal perfusion techniques to ensure complete removal of the tumour, protect the RA structures, and reconstruct the atrium [46,47]. The tricuspid valve and RA, as well as the LA and LV, should be carefully examined for multifocal tumours [38,41]. Regardless of the surgical approach, an ideal resection includes the tumour and the portion of the heart wall or atrial septum to which it is attached [38].

Ventricular myxomas are mainly removed via access through the atrioventricular valve [40,42,48]. If necessary, the tumour is resected through a direct ventricular incision, although this is an uncommon and least preferred approach [40,42,44]. It is not necessary to remove the full thickness of the ventricular wall. As with RA myxomas, ventricular myxomas also necessitate inspection of the heart cavities due to the high incidence of multiple tumours [48]. Every effort should be made to remove the tumour without fragmentation. Once the tumour is removed, the area should be generously washed and aspirated, and checked for any remaining fragments [41,47].

### 4.2. Minimally Invasive Approach (Right Anterolateral Mini-Thoracotomy)

The rapid development of minimally invasive procedures allows for less traumatic approaches to the surgical removal of heart tumours [49]. The first right anterolateral mini-thoracotomy (RAMT) used for myxoma resection was reported by Ko et al. [50] almost two decades ago. Since then, RAMT has been reported by many surgical centres as an alternative to MS. Currently, there are no standard criteria for using the MI approach in cardiac myxoma treatment [49,51].

Most often, patients not eligible for this surgery have previously undergone right thoracotomy or have peripheral vascular diseases, dense pleural adhesions, or severely decreased lung function [49,52].

In an MI surgical approach, after the induction of general anaesthesia, most often, a double-lumen endotracheal tube or a single tube with a bronchial blocker is placed to allow for single-lung ventilation [50,52,53]. Preoperative evaluation with TEE is then performed [50,52,53]. The patient should be placed in the supine position, with the right side of the body elevated [52,53]. Access to the chest is achieved through a right anterior mini-thoracotomy [50,52,53].

The pericardium is opened longitudinally, above the phrenic nerve. Pericardial stay sutures are placed to achieve adequate exposure of the heart. Arterial cannulation is performed usually via the femoral artery and the caval veins through the peripheral veins, usually the femoral, with eventually an additional cannula through a jugular vein [50,52,53].

The left atrium (LA) is accessed through a longitudinal incision in the Waterston’s groove, with a retractor employed to enhance visibility [52]. The myxoma is excised meticulously, ensuring the complete removal of all tumour tissue [52]. Complete resection is confirmed through direct inspection, and the mitral valve is evaluated for proper functionality [52,53]. The patient is weaned from CPB, and TEE is performed to confirm complete tumour resection, the absence of air embolism, and the lack of a residual interatrial shunt [53]. Pericardial and pleural drains are inserted. The thoracotomy incision is closed, and femoral vessel decannulation is performed to complete the procedure [53].

### 4.3. Robotic Assisted Approach

The robotic-assisted approach offers a promising therapeutic option for myxoma resection due to its excellent visualisation and exposure of the tumour. Multi-wristed robotic instruments enhance the ease and precision of excision compared to the long-shafted instruments used in minimally invasive cardiac surgery [54]. Despite being performed within a confined space, robotic excision provides superior manoeuvrability and a detailed view of the heart, thanks to magnified optics [55].

Surgical removal of a cardiac myxoma using the DaVinci© robotic platform follows similar stages to other minimally invasive cardiac surgery operations. Preparation involves the induction of general anaesthesia and the placement of a double-lumen endotracheal tube, indwelling urinary catheter, radial arterial line, and TEE probe [56,57]. The patient is positioned with the right chest elevated to about 30 degrees. Both groins and the chest are widely exposed [54,57]. Venous drainage and central catheters are percutaneously inserted into the right internal jugular vein [56,58].

The femoral vessels are cannulated either via a 2 cm transverse incision or percutaneously in the right groin using the Seldinger guidewire method, with the venous cannula guided under the TEE to the superior vena cava. Once connected to the heart– lung machine, extracorporeal circulation is initiated [54].

A small thoracotomy on the right side of the chest is made to facilitate the tumour’s removal. A soft tissue retractor is inserted, and a metal retractor may be added if needed. A traction suture may also be employed to improve exposure [54]. Ports for robotic arms are implanted through small skin incisions. CO_2_ is insufflated to facilitate exposure and lower the risk of air embolis ation.

An incision is made in the pericardium, anterior to the right phrenic nerve [56].

Antegrade cardioplegia is given either through a needle in the ascending aorta or through an endo-balloon.

For left atrial myxomas, excision is typically performed through a left atriotomy with Waterston’s groove visualisation or via a right atriotomy with an atrial septal incision [54,56]. For left ventricular myxomas, an additional retractor may be placed over the mitral valve [55]. Right atrial myxomas are usually resected via right atriotomy on the beating heart without aortic occlusion. Right ventricular myxomas are excised through the tricuspid valve or a right ventriculotomy, if necessary [58].

Tumour excision involves complete dissection, ensuring no fragmentation within the pleural space. Auxiliary sutures and an Endopouch bag are used to extract the tumour through the service port [56,59]. Large atrial myxomas may require vacuum-extractor devices for removal [60]. Iatrogenic defects or residual cavities in the heart wall are sutured or repaired using bovine pericardial patches if needed. After the removal of the tumour, the heart is deaired, and the atriotomy is closed [56].

Robotic myxoma excision is associated with shorter postoperative hospital stays (3–4 days) and recovery times (2–3 weeks) [60].

## 5. Comparison of the Surgical Approaches for Myxoma Resection

While median sternotomy (MS) has been the standard procedure for treating cardiac myxoma, minimally invasive (MI) surgical approaches are more and more often being used as the primary surgical approach in many cardiac surgery centres. Researchers have compared these techniques, especially focusing on mortality and morbidity outcomes.

A review of 13 articles provided data for the comparisons between these surgical strategies.

### 5.1. Median Sternotomy vs. Minimally Invasive Approach

Synthee et al. [61] recently published a comparative analysis of the short-term outcomes between right anterolateral mini-thoracotomy and median sternotomy for the resection of isolated left atrial myxomas in 28 patients, evenly distributed between the two groups. Preoperative characteristics, such as age, body mass index (BMI), and New York Heart Association (NYHA) classification, were comparable. The study revealed significant intraoperative and postoperative differences. In the MS group, the average CPB time was shorter (73.65 ± 14.3 min) compared to the RAMT group (94.29 ± 20.6 min), as was the aortic cross-clamp time (39.36 ± 13.15 min in MS vs. 51.14 ± 16.22 min in RAMT). However, the RAMT group showed significantly better postoperative outcomes, including a shorter intubation time (5.93 ± 1.73 vs. 11.43 ± 2.87 h), shorter ICU stays (30.34 ± 8.25 vs. 68.20 ± 20.93 h), and shorter hospital stays (5.71 ± 0.91 vs. 8.93 ± 2.46 days), along with lower pain scores (VAS 3.21 ± 1.81 vs. 5.00 ± 0.88) (*p* < 0.05). While complication rates were lower in the RAMT group (0% vs. 14.3% in MS), the difference was not statistically significant. The findings suggest that MI approaches like RAMT offer faster recovery times and fewer complications compared to traditional sternotomy for left atrial myxoma excision [61].

Mubarak et al. [62] conducted a retrospective study of 50 patients comparing RAMT (20 patients) and standard MS (30 patients) for atrial myxoma resection. Both approaches were found to be safe, with no perioperative deaths or conversions from thoracotomy to sternotomy. The key findings included significantly shorter ICU stays (1 ± 0.5 vs. 2 ± 1.5 days, *p* = 0.045), reduced chest drainage (535 ± 160 vs. 775 ± 260 mL, *p* = 0.01), and shorter hospital stays (7.2 ± 1.6 vs. 15.3 ± 2.4 days, *p* = 0.003) in the mini-thoracotomy group. The mini-thoracotomy group also had lower blood transfusion rates (6% vs. 35%, *p* = 0.029) and better pain scores. There were no differences in recurrence or complications during a 3-year follow-up. The study concluded that the MI approach is a safe and effective alternative to MS, offering advantages such as reduced recovery time and enhanced patient satisfaction [62].

Ellouze et al. [63] conducted a comparative study to evaluate RAMT versus MS for cardiac myxoma resection. Of the 43 patients included, 20 underwent the MI approach and 23 underwent MS. The study revealed significantly reduced preoperative blood loss in the MI group, with an average of 106 mL ± 95 compared to 338 mL ± 270 in the MS group. Postoperative outcomes were more favourable in the MI group, with shorter ICU (1.65 ± 1.2 days) and hospital stays (5.7 ± 3 days) compared to the MS group. These findings suggest that the MI approach offers advantages in terms of reduced blood loss and improved recovery times following cardiac myxoma resection [63].

Dong et al. [52] retrospectively analysed 66 patients diagnosed with cardiac myxoma, of which 30 patients underwent the mini-thoracotomy approach and 36 patients underwent MS for myxoma resection. The study found no significant differences between the groups in terms of aortic cross-clamp time, CPB duration, mortality, or postoperative complications. However, the MI group had a significantly shorter ICU stay (29.2 ± 6.5 h vs. 43.5 ± 6.9 h, *p* < 0.001) and postoperative hospital length of stay (5 days vs. 8 days, *p* < 0.001). The total cost of treatment was also significantly lower in the MI group (27,000 RMB vs. 33,000 RMB, *p* < 0.001) [52].

Deng et al. [64] compared thoracoscopic surgery with traditional MS for atrial myxoma resection. Among 64 patients, 40 underwent thoracoscopic surgery, while 24 underwent MS. The MI approach was associated with shorter ICU stays (17.67 ± 4.95 vs. 49.88 ± 3.21 h), less blood loss (127.87 ± 48.84 vs. 275.00 ± 59.01 mL), and shorter hospitalis ations (9.97 ± 3.54 vs. 15.13 ± 1.06 days). However, it resulted in longer operation times (208.08 ± 23.98 vs. 170.00 ± 16.58 min), CPB times (125.13 ± 29.33 vs. 59.13 ± 5.74 min), and aortic cross-clamp times (45.04 ± 16.82 vs. 33.96 ± 16.35 min) [64].

Petersen et al. [65] compared RAMT and conventional MS for the resection of atrial myxomas in 63 patients. The MI approach was used in 29 patients, while 34 patients underwent MS. The tumour sizes and intraoperative parameters, including cross-clamp time and procedural duration, were similar between groups. The postoperative outcomes such as ventilation time, drainage volume, and ICU and hospital stays showed no significant differences. The complication rates were low, with two minor strokes in the MI group and no major complications in either group. The authors concluded that MI resection is a safe and effective alternative to MS, offering comparable outcomes with reduced surgical trauma and improved cosmetic results [65].

Lee et al. [66] compared the outcomes of RAMT and MS for cardiac myxoma resection in 146 patients. Of these, 83 underwent MS, while 63 were treated using the MI approach. The study reported no early mortalities in either group and similar tumour recurrence rates, with two recurrences in the MS group and none in the RAMT group during follow-up. The CPB and aortic cross-clamp times were significantly shorter in the MS group (*p* < 0.001 and *p* = 0.005, respectively). The MI group required fewer postoperative blood transfusions (26% vs. 51%, *p* = 0.004) and had a lower incidence of arrhythmias (14% vs. 30%, *p* = 0.025). No significant differences were observed in postoperative intubation time, ICU stays, or overall hospital stays. No conversions from mini-thoracotomy to MS were required, demonstrating the feasibility of the MI technique [66].

Selkane et al. [67] reviewed 40 cases of cardiac myxoma, of which 37 underwent MS and 3 underwent video-assisted mini-thoracotomy. The myxomas were located in the left atrium (LA) in 34 cases, the right atrium (RA) in 4 cases, and in both atria in 1 case. The tumour sizes ranged from 2 to 10 cm. Histological analysis confirmed the diagnosis in all cases. Three early postoperative deaths (7.5%) occurred due to severe complications: mitral ring rupture, cerebral tumour embolism, and septic shock. The immediate outcomes were favourable in the remaining 37 patients, with particularly positive results in the three treated with MI surgery, although the sample size was too small for definitive conclusions [67].

The results are summarised in Table 2.

### 5.2. Median Sternotomy vs. Robotic Assisted Approach

Liu et al. [68] compared conventional sternotomy and robotic-assisted approaches in a study of 94 patients undergoing left atrial myxoma resection. While both methods were safe and effective, the robotic approach led to significantly less postoperative drainage (330 vs. 105 mL, *p* < 0.001), shorter mechanical ventilation time (8 vs. 4.8 h, *p* = 0.005), and reduced hospitalis ation time (10 vs. 9 days, *p* = 0.04). However, there were no significant differences in operation time, ICU stays, or blood transfusion needs between the groups. The robotic approach, despite higher costs, offers enhanced recovery and can be considered a viable alternative to sternotomy [68].

Ersin Kadiroğullar et al. [69] found that robotic surgery for cardiac myxoma resulted in lower postoperative blood loss (240 vs. 110 mL, *p* = 0.001), reduced transfusion rates (31.3% vs. 66.7%, *p* = 0.022), and shorter hospital stays (4.3 ± 0.4 vs. 6 ± 2.4 days, *p* = 0.048), although the mean CPB (107.8 ± 52.7 vs. 58.3 ± 26.8, *p* = 0.001) and aortic cross-clamp times (58.7 ± 29.3 vs. 29.4 ± 12.2, *p* = 0.001) were shorter in the MS group. There were no major complications or conversions to sternotomy in the robotic group, and pain management was better in the patients undergoing robotic surgery. This study supports the view that robotic surgery may offer better postoperative outcomes [69].

Emmanuel Moss et al. [70] also confirmed that robotic-assisted surgery led to lower transfusion rates (13.3% vs. 46.2%, *p* = 0.004), shorter ventilation (4.3 ± 5.2 vs. 13.8 ± 34.8 h, *p* = 0.01) and ICU stays (26.1 ± 17.7 vs. 45.5 ± 47.7 h, *p* = 0.01), and reduced hospital stays (4.3 ± 1.8 vs. 5.8 ± 3.3, *p* = 0.01) compared to the sternotomy approach. There was no significant difference in major complications between the two approaches, suggesting that the robotic-assisted method may offer superior perioperative outcomes [70].

Ming Yang et al. [71] compared postoperative outcomes, including pain, quality of life (QoL), and length of sick leave. The robotic-assisted approach had shorter ventilation (7.1 ± 2.1 vs. 8.4 ± 3 h, *p* < 0.01) and intensive care time (2.7 ± 1 vs. 4.1 ± 3.6 days, *p* = 0.04), and a lower incidence of postoperative atrial fibrillation (4.1% vs. 18.2%, *p* = 0.04) compared to the sternotomy approach. The results also showed that robotically assisted surgery led to significantly better outcomes in various QoL dimensions, such as physical functioning, bodily pain, general health, and social functioning, compared to conventional surgery. Patients in the robotically assisted group reported less pain, minimal impact on daily life, and quicker return to work [71].

### 5.3. Minimally Invasive Approach vs. Robotic Assisted Approach

A study by Yanyi Liu et al. [57] compared thoracoscopic (minimally invasive) and robotic approaches for myxoma resection. The robotic approach had shorter operative and CPB times (97.9 ± 24.5 vs. 118.27 ± 38.8 min, *p* = 0.035), as well as less ICU (20 vs. 39 h, *p* = 0.006) and ventilation time (6 vs. 13 h, *p* = 0.035), but the thoracoscopic approach had lower operating room and total hospital costs. Both methods had similar postoperative complications, suggesting that both techniques are viable for cardiac myxoma surgery, with the choice depending on the available resources and patient or surgeon preferences [57].

## 6. Conclusions

**Figure 2:**
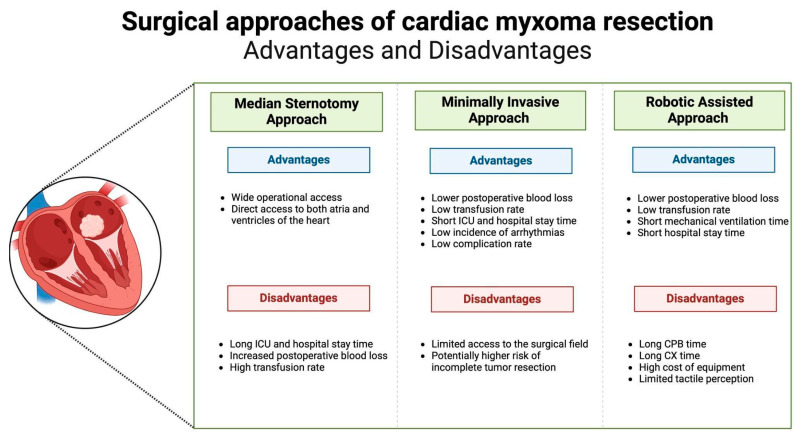
Advantages and disadvantages of different surgical approaches of cardiac myxoma resection.

The most commonly used technique for cardiac myxoma resection remains median sternotomy, offering reliable surgical access and short CPB times. However, it is associated with longer ICU stays, in-hospital stays, and greater perioperative blood loss. Minimally invasive surgery provides an alternative, with reduced recovery time, lower blood loss, and better postoperative satisfaction. However, it presents challenges such as restricted surgical exposure, which can increase the risk of incomplete tumour resection and longer CPB and cross-clamp times. Advantages and disadvantages of different surgical approaches of cardiac myxoma resection were summarised in Figure 2.

Robotic-assisted surgery has shown improved QoL outcomes, including reduced postoperative pain and better physical functioning. However, it is more expensive and requires specialised equipment, which limits its widespread adoption. Despite the higher costs, robot-assisted surgery offers enhanced recovery outcomes and can be considered for patients who are suitable candidates. All of the studies have limitations associated with the size of the study group.

Ultimately, the choice of surgical approach should be tailored to the individual patient’s condition, tumour characteristics, surgeon expertise, and the local availability of the surgical equipment. The median sternotomy approach is mainly considered for patients with large or complex tumours or those with the potential for embolic complications [72]. On the other hand, minimally invasive techniques are preferred for younger, lower-risk patients with isolated, accessible myxomas where a less invasive procedure may offer recovery benefits [73].

With the increasing development of minimally invasive and robotic techniques, more studies are needed to assess the long-term outcomes and efficacy of these methods in cardiac myxoma resection.

## Figures and Tables

**Table 1:** Frequency of cardiac myxoma symptoms.

Symptoms	Frequency (%)
Cardiac symptoms:	
Dyspnoea	70
Heart failure	67
Embolic incidents cerebral embolic incidents peripheral embolic incidents	30
	66
	33
Postural syncope	20
Cardiac arythmias	20
Fever	19
Weight loss	17
Chest pains	10
Systemic symptoms: leukocytosis accelerated erythrocytes sedimentation polycytaemia haemolytic anaemia thrombocytopenia/thrombocytosis elevated CRP levels hypergammaglobulinaemia neutrophilia elevated interleukin-6	100

**Table 2:** Comparison of median sternotomy and minimally invasive approach for myxoma resection.

	CBP Time, min			Ventilation Time, hours			ICU Stay Time, days			Hospital LOS, days		
	MS	MI	*p* Value	MS	MI	*p* Value	MS	MI	*p* Value	MS	MI	*p* Value
Synthee et al. [61]	73.7 ± 14.3	94.3 ± 20.6	0.005	11.4 ± 2.9	5.9 ± 1.7	<0.001	2.8 ± 0.9	1.3 ± 0.3	<0.001	8.9 ± 2.5	5.7 ± 0.9	<0.001
Mubarak et al. [62]	87 ± 25	110 ± 32	0.15	10.5 ± 1.1	9.2 ± 2.3	0.37	2 ± 1.5	1 ± 0.5	0.045	15.3 ± 2.4	7.2 ± 1.6	0,003
Ellouze et al. [63]	54.3 ± 25	64.3 ± 18	0.141	5 ± 2.4	3.9 ± 1.4	0.07	2.3 ± 1.8	1.7 ± 1.2	0.178	7.1 ± 2	5.7 ± 3	0.09
Dong et al. [52]	83.6 ± 8.4	87.8 ± 10.4	0.076	-	-	-	1.8 ± 0.3	1.2 ± 0.3	<0.001	8	5	<0.001
Deng et al. [64]	59.1 ± 5.7	125.1 ± 29.3	<0.001	6 ± 2.5	4.8 ± 2.1	<0.01	2.1 ± 1.3	0.7 ± 0.2	<0.001	15.1 ± 1.1	10 ± 3.5	<0.01
Petersen et al. [65]	-	-	-	5.4 ± 3	5.8 ± 3.7	0.718	2.6 ± 2	1.8 ± 1.2	0.078	7.2 ± 1.9	6.7 ± 2.9	0.409
Lee et al. [66]	73.4 ± 32	94.4 ± 33	<0.001	10.7 ± 18	6.7 ± 3.3	0.053	2.8 ± 9.1	1.1 ± 0.4	0.168	11.7 ± 24.7	7.1 ± 6.6	0.156

MS = median sternotomy; MI = minimally invasive approach; LOS = length of stay.

## Data Availability

Not applicable.

## Abbreviations

CPB	cardiopulmonary bypass
RA	right atrium
LA	left atrium
RV	right ventricle
LV	left ventricle
MS	median sternotomy
RAMT	right anterolateral minithoracotomy
MI	minimally invasive
TEE	transoesophageal echocardiography
ICS	intercostal space
AAL	anterior axillary line
BMI	body mass index
NYHA	New York Heart Association
ICU	intensive care unit
QoL	Quality of Life
